# Synthesis, In Silico Study, and Anti-Cancer Activity of Thiosemicarbazone Derivatives

**DOI:** 10.3390/biomedicines9101375

**Published:** 2021-10-01

**Authors:** Belay Zeleke Sibuh, Piyush Kumar Gupta, Pankaj Taneja, Sonia Khanna, Paratpar Sarkar, Sanya Pachisia, Abrar Ali Khan, Niraj Kumar Jha, Kamal Dua, Sachin Kumar Singh, Sadanand Pandey, Petr Slama, Kavindra Kumar Kesari, Shubhadeep Roychoudhury

**Affiliations:** 1 Department of Biotechnology, School of Engineering and Technology (SET), Sharda University, Knowledge Park III, Greater Noida 201310, Uttar Pradesh, India; belayzeleke63@yahoo.com (B.Z.S.); pankaj.taneja@sharda.ac.in (P.T.); niraj.jha@sharda.ac.in (N.K.J.); 2 Department of Life Sciences, School of Basic Sciences and Research (SBSR), Sharda University, Knowledge Park III, Greater Noida 201310, Uttar Pradesh, India; dr.piyushkgupta@gmail.com; 3 Department of Chemistry and Biochemistry, School of Basic Sciences and Research (SBSR), Sharda University, Knowledge Park III, Greater Noida 201310, Uttar Pradesh, India; paratpar.sarkar@sharda.ac.in; 4 Department of Chemistry, University of Delhi, New Delhi 110007, Delhi, India; sanyapachisia@gmail.com; 5 Department of Biotechnology, Indian Institute of Technology, Chennai 600036, Tamilnadu, India; abrar1588@gmail.com; 6 Discipline of Pharmacy, Graduate School of Health, University of Technology Sydney, Sydney, NSW 2007, Australia; Kamal.Dua@uts.edu.au; 7 School of Pharmaceutical Sciences, Lovely Professional University, Phagwara 144001, Punjab, India; sachin.16030@lpu.co.in; 8 Department of Chemistry, College of Natural Science, Yeungnam University, 280 Daehak-Ro, Gyeongsan 38541, Gyeongbuk, Korea; sadanand.au@gmail.com; 9 Department of Animal Morphology, Physiology and Genetics, Faculty of AgriSciences, Mendel University in Brno, 61300 Brno, Czech Republic; petr.slama@mendelu.cz; 10 Department of Bioproducts and Biosystems, School of Chemical Engineering, Aalto University, 00076 Espoo, Finland; kavindra.kesari@aalto.fi; 11 Department of Life Science and Bioinformatics, Assam University, Silchar 788011, Assam, India

**Keywords:** molecular docking, in silico ADMET, 3-Methoxybenzaldehyde thiosemicarbazone, 4-Nitrobenzaldehyde thiosemicarbazone, cancer, B16-F0 melanoma, MCF-7

## Abstract

Thiosemicarbazones are known for their biological and pharmacological activities. In this study, we have synthesized and characterized 3-Methoxybenzaldehyde thiosemicarbazone (3-MBTSc) and 4-Nitrobenzaldehyde thiosemicarbazone (4-NBTSc) using IR, ^1^HNMR and ^13^C NMR. The compound’s in vitro anticancer activities against different cell lines were evaluated. Molecular docking, Insilco ADMET, and drug-likeness prediction were also done. The test compounds showed a comparative IC_50_ and growth inhibition with the standard drug Doxorubicin. The IC_50_ ranges from 2.82 µg/mL to 14.25 µg/mL in 3-MBTSc and 2.80 µg/mL to 7.59 µg/mL in 4-NBTSc treated cells. The MTT assay result revealed, 3-MBTSc inhibits 50.42 and 50.31 percent of cell growth in B16-F0 and EAC cell lines, respectively. The gene expression showed that tumor suppressor genes such as PTEN and BRCA1 are significantly upregulated in 7.42 and 5.33 folds, and oncogenes, PKC, and RAS are downregulated −7.96 and −7.64 folds, respectively in treated cells. The molecular docking performed on the four targeted proteins (PARP, VEGFR-1, TGF-β1, and BRAF^V600E^) indicated that both 4-NBTSc and 3-MBTSc potentially bind to TGF-β1 with the best binding energy of −42.34 Kcal/mol and −32.13 Kcal/mol, respectively. In addition, the test compound possesses desirable ADMET and drug-likeness properties. Overall, both 3-MBTSc and 4-NBTSc have the potential to be multitargeting drug candidates for further study. Moreover, 3-MBTSc showed better activity than 4-NBTSc.

## 1. Introduction

Cancers arise due to the accumulation of mutations in essential genes that can induce cellular alteration and malignant transformation [1]. Cancer affects all ages and all social classes, and it was the second most common cause of death worldwide in 2018, with 18.1 million new cases and 9.6 million deaths in the same year. There will be about 29–37 million cancer cases by 2040 [2]. Breast cancer is the most common malignancy diagnosed (11.7%) in 2020 among women worldwide. Breast cancer affected 2.26 million women in 2020, killing an estimated 684 996 of them and accounting for approximately 6.9 percent of all cancer deaths [3,4]. Furthermore, there has been an increasing incidence of non-melanoma and melanoma skin cancers over the past few decades [5]. Non-melanoma skin cancer accounted for 6.2% of all cancer cases in 2020 and was the fifth most common type of cancer, affecting 1.198 million people in 2020 [3,6].

The mitogen-activated protein kinase/extracellular signal regulated kinase (MAPK/ERK) signaling pathway (also known as the RAS-RAF-MEK-ERK pathway) mediates cellular responses to extracellular stimuli [1,7]. BRAF is a serine/threonine protein kinase activating the MAP kinase/ERK-signaling pathway [8]. Different types of human cancers are associated with mutations in MAPK/ERK signaling pathways and the B-RAF proto-oncogene (BRAF) gene [7,9]. MAPK/ERK signaling is essential for melanoma development and progression [10]. Hence, the RAS/RAF/MEK/ERK pathway has been reported to be activated in over 80% of all cutaneous melanomas [11] and about 50% of melanomas harbor activating BRAF mutations [8]. The most common mutations in the BRAF gene encode the V600E mutant (class I) caused by an amino acid substitution at position 600 in the BRAF protein, from valine (V) to a glutamic acid (E), as a result of the transversion c.1799T>A in exon 15 [1]. This mutation causes continuous activation and signal transduction of BRAF kinase regardless of external stimulus leading to enhanced cell proliferation and invasion [7].

TGF-β is a large growth factor family member including activins/inhibins and bone morphogenic proteins (BMPs) that play essential roles in cell growth, differentiation, apoptosis, migration, normal skin homeostasis, epidermal stem cells regulation, angiogenesis, and inflammation as well [12,13]. The transforming growth factor-β (TGF- β) has attracted much attention as a therapeutic target since it plays an important and pleiotropic role in melanoma progression [14,15].

Genomic stability is maintained by intricate and highly complex biochemical pathways regulated by a multitude of proteins. When one or more of these pathways is disrupted by biological, physical, or chemical means, cells are at risk of mutagenesis and, as a result, carcinogenesis [16,17]. The DNA damage response (DDR) pathway coordinates the identification, signaling, and repair of DNA damage caused by different factors. Poly (ADP-ribose) polymerase (PARP) is the best-known element of the DDR [17]. The poly (ADP-ribose) polymerases (PARPs) are DNA-binding enzymes that play a vital role in DNA repair. The use of PARP inhibitors is a rational therapeutic approach to cause apoptosis in cancer cells lacking DNA repair pathways [18,19]. Poly (ADP-ribose)-polymerase inhibitors (PARPi) are of particular interest in treating hereditary breast cancers occurring in patients with germline BRCA1/2 (gBRCA1/2) mutations [20]. Indeed, the US Food and Drug Administration (FDA) has recently approved PARP inhibitors to treat breast and ovarian cancers. Despite the proven efficacy of these agents in improving progression-free survival, cancer cells inevitably developed resistance. The primary and secondary resistance make long-term use of PARP inhibitors difficult [16,21]. Hence finding a better inhibitor with less resistance from cancer cells is essential in the fight against cancer.

Tumor angiogenesis is an essential process for tumor growth and metastasis [22,23], which is controlled by vascular endothelial growth factors (VEGFs) and their receptors (VEGFRs) [24]. Vascular endothelial growth factor (VEGF) is a mitogen that plays a crucial role in angiogenesis and lymphangiogenesis [25]. Vascular endothelial growth factor (VEGF) promotes breast cancer progression by inducing angiogenesis via VEGF receptors on endothelial cells but also signals directly through receptors such as VEGFR-1 (Flt-1) expressed on tumor cells [26]. In addition, reduced or lost VEGFR1 expression may serve as a marker for poor prognosis in patients with breast cancer, who might not optimally benefit from endocrine therapy [24]. Overall, VEGF is involved in tumor survival by inducing tumor angiogenesis and increasing chemoresistance through autocrine signaling [25].

A high-quality drug candidate should exhibit a strong interaction, adequate efficacy against the therapeutic target, and appropriate ADMET properties at a therapeutic dose [27]. For this purpose, computational approaches such as molecular dynamics simulations, drug-likeness prediction, in silico ADMET analysis, molecular docking, etc., help identify potential drugs/molecules from various databases and reduce time and experimental costs in drug discovery [28]. Molecular docking could effectively predict the binding mode and binding energy of the protein-ligand complexes [29] and, ADMET prediction used to determine the absorption, distribution, metabolism, excretion, and toxicity (ADMET) property of a potent molecule [27].

Thiosemicarbazones are an important class of compounds known for their versatile biological and pharmacological activities. They are valuable intermediates in synthesizing pharmaceutical and bioactive materials and are thus widely used in medicinal chemistry [30,31]. In recent years, derivatives of thiosemicarbazones have demonstrated a broad range of biological and pharmacological activities (viz. antifungal, anticancer, antimicrobial, and antiviral) [32,33,34,35,36,37,38,39,40,41]. In line with this, we found no research on the in silico ADMET and drug-likeness prediction, as well as the anticancer activity of the same compound presented in our study. However, there have been few studies on their structure [42,43], antifungal activity [44], and anti-parasite activity [45,46]. Therefore, this study aimed to synthesize, characterize, evaluate the in-vitro anticancer activity, perform molecular docking, and predict the ADMET and drug likeness property of 3-Methoxybenzaldehyde thiosemicarbazone and 4-Nitrobenzaldehyde thiosemicarbazone.

## 2. Materials and Methods

### 2.1. Materials

Analytical grade chemicals obtained from Sigma-Aldrich (Sigma Aldrich, St. Louis, MI, USA) were used to synthesize the ligands steps presented below. Structural analysis was done through FTIR, ^1^H-NMR, and ^13^C-NMR spectral studies.

### 2.2. Molecular Docking

Molecular docking calculations were carried out using Patch dock server-Bioinfo 3D [47]. Four targeted proteins, BRAF, TGF-β1, PARP, and VEGFR-1 were selected based on their role in different cellular signaling pathways for the development and progression of breast and skin cancers. BRAF^V600E^ is a common mutation in BRAF protein of melanoma cells [8]. Pro-Transforming Growth Factor-beta 1 (TGF-β1) plays a pleiotropic role in melanoma progression [14,15] and it is also an important regulator of normal mammary gland development as well as it leads to the progression of breast cancers [48]. While poly (ADP-ribose) polymerase (PARP) is a nuclear enzyme that facilitates DNA repair and play an imperative role in germline mutations [20]. Further, Vascular Endothelial Growth Factor Receptor-1 (VEGFR-1) plays a significant role in tumor-associated angiogenesis, tissue infiltration, and metastasis development [49]. All structures of BRAF^V600E^ (PDB ID: 6V34), TGF-β1 (PDB ID: 3RJR), PARP (PDB ID: 1A26), and VEGFR-1 (PDB ID: 5ABD) were downloaded as PDB file from Protein Data Bank database (https://www.rcsb.org/. Last accessed 12 February 2021). All protein structures were cleaned of inhibitors and water molecules to avoid their interference during docking. Chem Sketch was used to draw and clean the structures of the synthesized compounds (3-MBTSc and 4-NBTSc). Then, the mol file was generated and converted to PDB format. After that, both compounds were docked to the active sites of the enzymes. Patch dock generated 20 best compound-enzyme interactions in various positions; then, using Fire dock, 10 more refined results were selected based on global energy (binding energy of the solution), attractive and repulsive van der Waals energy, atomic contact energy, geometric shape complementarity score, and approximate interface area.

### 2.3. Drug-Likeness Prediction

Drug-likeness evaluates the likelihood of a molecule becoming an oral drug in terms of bioavailability [50]. The drug-likeness was predicted using the OSIRIS Property Explorer (http://www.organic-chemistry.org/prog/peo/. Last accessed on 20 July 2021). The OSIRIS Property Explorer uses chemical structures and calculates the drug-related properties whenever a structure is valid [51]. Similarly, SwissADME predicts the drug-likeness by filtering and excluding molecules with incompatible properties with an acceptable pharmacokinetic profile. SwissADME uses five different rule-based filters, with diverse ranges of properties inside of which the molecule is defined as drug-like [50]. The Lipinski filter (Pfizer) rule of five is the pioneer in this regard that characterize small molecules based on physicochemical property profiles which include molecular weight (MW) less than 500, N or O ≤ 10 (H-bond acceptors), NH or OH ≤ 5 (H-bond donors) [52,53]. Hence, we analyzed Lipinski’s rule of screening medicinal chemistry property of the test compound using SwissADME (http://www.swissadme.ch/index.php. Last accessed on 20 July 2021) [50].

### 2.4. ADMET Prediction

ADMET stands for absorption, distribution, metabolism, excretion, and toxicity of the test compound. The molecular structure of the test compound was submitted to the admetSAR server (http://lmmd.ecust.edu.cn/admetsar1. Last accessed on 20 July 2021) [54]. The sever uses 47 different models to predict more than 50 ADMET properties of the query molecules [54,55]. The different pharmacokinetic and pharmacodynamic properties predicted in our study include blood-brain barrier penetration (BBB), human intestinal absorption (HIA), Caco-2 permeability, cytochrome P450 solubility, cytochrome P (CYP) inhibitory promiscuity, renal organic cation transportation, carcinogenesis, Ames toxicity, and rat acute toxicity.

### 2.5. Synthesis of 3-Methoxybenzaldehyde Thiosemicarbazone (3-MBTSc) 

3-Methoxybenzaldehyde thiosemicarbazone (C_9_H_11_N_3_OS; (2*E*)-2-(3-methoxybenzylidene) hydrazine carbothioamide) (3MBTSc) was prepared by using the previous procedure with slight modifications [43]. It was prepared by refluxing a mixture of thiosemicarbazide (2.0 g, 0.01 mmol) and 3-Methoxybenzaldehyde (2.67 mL, 0.01 mmol) in a mixture of 20 mL distilled water and 20 mL methanol as the solvent medium in the presence of glacial acetic acid (2–3 mL). The reaction mixture was refluxed for about 3–4 h. After cooling, the yellow crystalline solid separated was filtered, washed, and dried. The yield of the compound was 2.16 g and the obtained compound was insoluble in CHCl_3_, CCl_4_, CH_3_CN, H_2_O, ethanol, and methanol but it was soluble in DMSO and DHF/THF. The following characterization was carried out to confirm the synthesis of 3-Methoxybenzaldehyde thiosemicarbazone by FTIR (cm^−1^): v(NH_2_) 3403s; 3287s; v(-NH) 3150s; 1599s v(C=N); v(C=S) 1246s, 836s, ^1^H-NMR (DMSO-d^6^, ppm): 11.40 (s, 1H); 8.19 (s, 2H); 6.95–7.44 (m, ar-H); 3.80 (s, 3H), and ^13^C-NMR (DMSO-d^6^, ppm): 178.9 (s, C=S), 55.76 (s, OCH3), 111-143 (m-Ph-C), 159.6 (s, C=N). 

### 2.6. Synthesis of 4-Nitrobenzaldehyde Thiosemicarbazone (4-NBTSc)

4-Nitrobenzaldehyde thiosemicarbazone ((2*E*)-2-(4-nitrobenzylidene) hydrazine carbothioamide) (4NBTSc) was synthesized using earlier reported methods with slight modifications [46,56]. A mixture of 1.51 g (0.01 mmol) of 4-Nitrobenzaldehyde, and 0.914 g (0.01 mmol) thiosemicarbazide was stirred for 2 h using 15 mL of methanol and 15 mL of distilled water as a solvent medium. The resulting yellow solution was allowed to cool. Then, a yellow crystalline solid separated was filtered, washed with diethyl ether and dried. The yield of the compound was 2.30 g and the obtained compound was insoluble in CHCl_3_, CCl_4_, CH_3_CN, H_2_O, ethanol, and methanol but it was soluble in DMSO and DHF/THF. The following characterization was carried out to confirm the synthesis of 4-Nitrobenzaldehyde thiosemicarbazone by FTIR (cm^−1^): v(NH_2_) 3499m; 3373s; v(-NH) 3197s; 1588s; 1343s (v(NO2); v(C=N); v(C=S) 1137s, 750s, ^1^H-NMR (DMSO-d^6^, ppm): 11.66 (s, 1H); 8.38 (s, 2H); 8.32–8.03 (m, ar-H), and ^13^C-NMR (DMSO-d^6^, ppm): 179 (s, C=S), 128-141 (m-Ph-C), 148.06(s, C=N), 124.23 (s, CH).

### 2.7. Cell Lines and Cell Culture

The in vitro anticancer activities of the synthesized compounds were conducted using different cancerous and non-cancerous cell lines *viz* B16-F0 (Mouse melanoma), mouse Ehrlich Ascites Carcinoma (EAC), MCF-7 (Human breast cancer), MCF-10A (Human breast epithelial cells), and NIH/3T3 (Mouse fibroblast cells). All cell lines were procured from the National Centre for Cell Science, Pune, India. The procured cells were cultured in a six-well culture plate with Dulbecco’s Modified Eagle’s Medium (DMEM) supplemented with 10% fetal bovine serum in a humid CO_2_ incubator with 5% carbon dioxide at 37 °C.

### 2.8. Anti-Cancer Potential and Cytotoxicity Analysis

The anti-cancer effect and cytotoxicity of both 3-MBTSc and 4-NBTSc compounds were evaluated against different cancerous (MCF-7, EAC, B16-F0 cells) and noncancerous cells (MCF-10A, NIH-3T3 cells) using MTT test [57,58]. In brief, the stock solutions of both the tested compounds were prepared by dissolving 1 mg of each compound in 1 mL of DMSO separately. Then, four different concentrations (5, 10, 12, and 20 µg/mL) of both compounds were used during the test along with the negative control. Also, 10 μg/mL of doxorubicin (Dox) was used as the positive control during the experiment. Both untreated and treated cells were incubated for next 24 and 48 h. Then, all the steps during the whole experiment were carried out by our earlier reported procedure [59,60,61]. Later, the absorbance values were measured by an ELISA reader (Biorad, Hercules, CA, USA) at 570 nm wavelength using secondary wavelength as a reference at 620 nm and the percentages of cell viability was calculated using the following formula [59,60,61].

$$\mathrm{Cell}\;\mathrm{viability}\;(\%)=\;\frac{\mathrm{Mean}\;\mathrm{absorbance}\;\mathrm{of}\;\mathrm{treated}\;\mathrm{cells}}{\mathrm{Mean}\;\mathrm{absorbance}\;\mathrm{of}\;\mathrm{untreated}\;\mathrm{cells}}\;\times \;100$$

Based on the cell viability data, the IC_50_ values of both compounds were calculated on different cancer cells by plotting their corresponding dose-responsive graphs.

### 2.9. Gene Expression Analysis

Total mRNA was isolated and extracted by Trizol reagent as described in an earlier method [62,63]. RevertAid First Strand cDNA synthesis kit (Thermo Fisher Scientific, Waltham, MA, USA) was used to synthesize cDNA in a total reaction volume of 20 μL containing oligo-dT primers, RiboLock RNase inhibitor, and reverse transcriptase. Gene-specific primers were used to amplify cDNAs in RT-qPCR machine (Applied Biosystems, Waltham, MA, USA).

### 2.10. Statistical Analysis

The obtained data were statistically analyzed. The descriptive analysis was done and the IC_50_ values were calculated by fitting the sigmoidal dose responsive graph using GraphPad Prism (GraphPad Software, Inc., La Jolla, CA, USA). Also, two-way ANOVA and Tukey multiple comparison tests were used. The statistical significance between the control and treated cells was considered when a = * *p* < 0.05, b = ** *p* < 0.01, c = *** *p* < 0.001, d = **** *p* < 0.0001

## 3. Results

### 3.1. Molecular Docking

**Figure 1:**
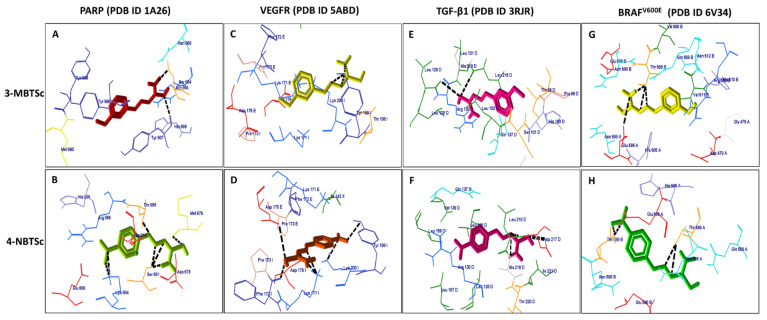
Interaction of 3-MBTSc and 4-NBTSc with (**A**,**B**) PARP: Poly (ADP-ribose) polymerase; (**C**,**D**) VEGFR-1: Vascular endothelial growth factor receptor-1 (**E**,**F**) TGF-β1: pro- transforming growth factor beta 1 (**G**,**H**) BRAF^V600E^. (**A**) **3-MBTSc** (Red) formed one hydrogen bonding with Tyrosine 907 (Midnight blue) and Serine 854 (Royal). (**B**) **4-NBTSc** (green) interacted with four amino acids, Serine 681(Honey), Lysine 684 (light blue), Threonine 866 (Royal) and Methionine 675 (Yellow). (**C**) **3-MBTSc** (Yellow) made three hydrogen bonds with Tyrosine 199 (Midnight blue) amino acid. (**D**) **4-NBTSc** (Bronze) has interacted with Proline 173 (Cantaloupe), Aspartate 175 (Red) and Tyrosine 199 (Midnight blue) with one hydrogen boding and lysine171 and Lysine 200 (light blue) in three hydrogen bonds. (**E**) **3-MBTSc** (Pink) formed two hydrogen bonds with Leucine amino acid (Leu 218 and Leu 129) (Green). (**F**) Amino acid- Alanine 219 (White), Leucine 218(Green), Aspartate 217 (Red) and Serine 228 are involved in one hydrogen bond with the synthesized compound **4-NBTSc** (Carmine). (**G**) **3-MBTSc** (Yellow) formed one hydrogen bond with Glutamate 586 (Red) and three hydrogen bonds with Threonine 589 (Royal). (**H**) The test compound **4-NBTSc** (Green) formed four hydrogen bonds with Threonine 589 (Royal) amino acid.

To rationalize the therapeutic potential of the test compound and determine their binding modes, a molecular docking study was performed using Patch dock server-Bioinfo 3D [47]. The best binding mode of docked compounds, 3-MBTSc and 4-NBTSc with different targeted proteins are presented in Figure 1 and Table 1. Based on the output we synthesized and tested the in vitro anticancer activity of the 3-MBTSc and 4-NBTSc in breast and skin cancer cell line. 

### 3.2. Drug Likeness Prediction

Drug-likeness evaluates the chance for a molecule to become an oral drug with respect to bioavailability [50]. The drug-likeness and medicinal chemistry properties of the test compounds were predicted using OSIRIS Property Explorer (http://www.organic-chemistry.org/prog/peo/. Last accessed on 20 July 2021) and SwissADME (http://www.swissadme.ch/index.php. Last accessed on 20 July 2021) [50]. The drug-likeness prediction result is presented in Table 2. 4-NBTSc showed a lower Clog P than 3-MBTSc, indicating better hydrophilicity and, thus, good absorption and permeation. A log *S* value indicates solubility; the higher the log *S* value, the lower the solubility which would reduce the absorption [51]. In terms of solubility, 4-NBTSc is more soluble in blood than 3-MBTSc. Similarly, a lower TPSA is associated with higher membrane permeability, and thus a lower TPSA is favorable for drug-like properties [64]. According to Lipinski’s rule of five, TPSA ranges from 0–140 Å^2^, and the studied compounds have TPSA in the acceptable range with 3-MBTSc having a lower value than 4-NBTSc. The two compounds did not violate the Lipinski rule of five with respect to molecular weight, TPSA, number of rotatable bonds, H-bond acceptors, H-bond donors [52,53]. The bioavailability score in SwissADME predicts the probability of a compound having at least 10% oral bioavailability in rat or measurable Caco-2 permeability based on total charge, TPSA, and violation to the Lipinski filter [50]. Both 3-MBTSc and 4-NBTSc showed a good and similar bioavailability score (Table 2 and Figure S1).

### 3.3. ADMET Properties

**Figure 2:**
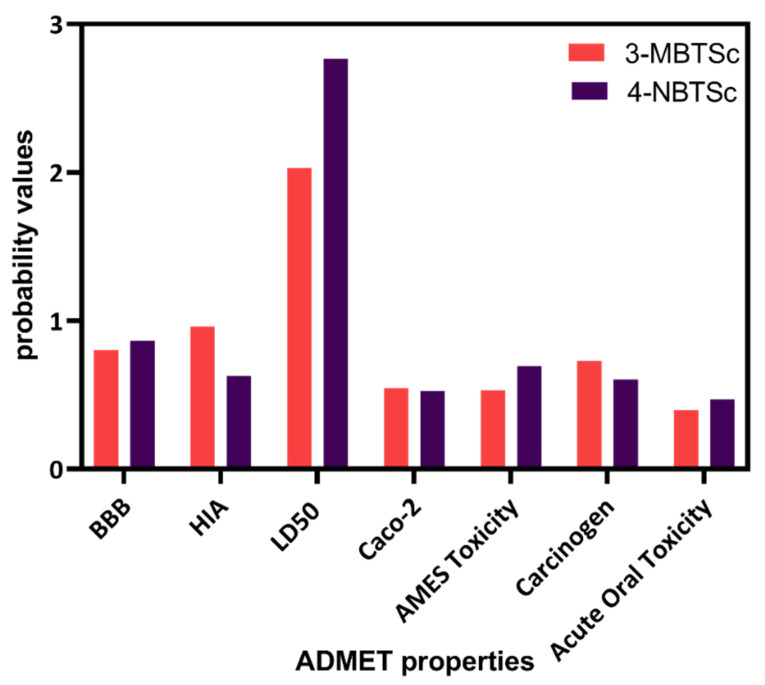
Comparative ADMET properties of 3-MBTSc and 4-NBTSc compounds.

The ADMET properties of the test compounds are predicted using admetSAR (http://lmmd.ecust.edu.cn/admetsar1. Last accessed on 20 July 2021) [54] and presented in Figure 2 and Table S3. Blood-brain barrier (BBB) penetration, human intestinal absorption (HIA), Caco-2 cell permeability, and AMES toxicity along with LD50 and additional ADMET properties were calculated. A blood-brain barrier and human intestinal absorption value closer to 1 represents better permeability and absorption through BBB and intestine [65]. Both 3-MBTSc and 4-NBTSc have high intestinal absorption and BBB permeability. When predicting the efflux by P-glycoprotein (P-gp), they prove to be both non-substrate and non-inhibitor. Also, both the test compounds exhibit non-substrate and non-inhibitor behavior on most of the CYP450 isoforms. AMES toxicity and carcinogenicity test helps to identify a potential mutagenic and cancer-causing molecule. Lethal Dose 50 (LD50) is the quantity of a drug that can kill 50% of the test organisms’ population [65]. Both the test compounds are non-carcinogenic and have a good LD50.

### 3.4. Synthesis and Characterization of 3-MBTSc and 4-NBTSc Compounds

**Scheme 1:**
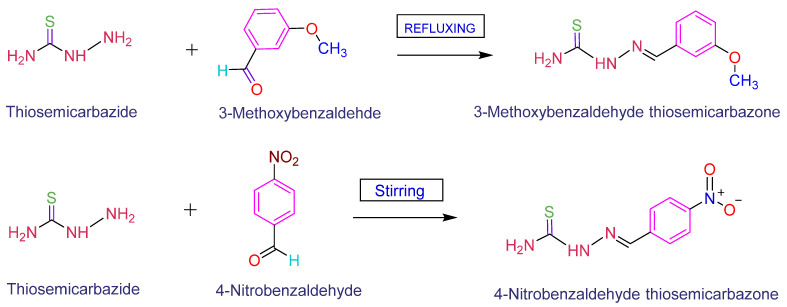
Schematic representation of the synthesis of 3-MBTSc and 4-NBTSc compounds.

**Figure 3:**
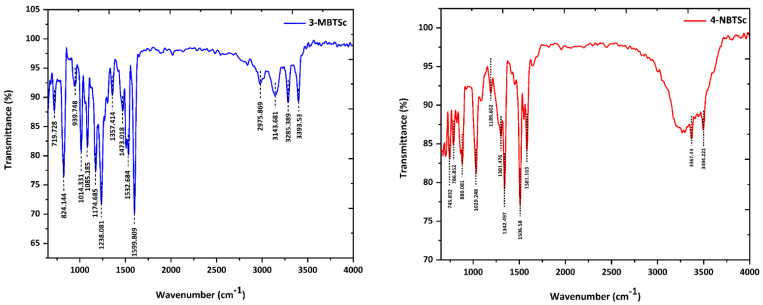
FTIR spectra of 3-MBTSc and 4-NBTSc compounds in the wavenumber range of 600–4000 cm^−1^.

3-MBTSc and 4-NBTSc compounds were synthesized by the following steps as illustrated in Scheme 1. The chemical structural analysis was carried out by FT-IR, ^1^H-NMR and ^13^C-NMR spectral studies and it has confirmed the purity of the synthesized compounds. As depicted in Figure 3, the vibrational peaks between 3208–3499 cm^−1^ and 3147–3197cm^−1^ correspond to the *v*(-NH_2_) and *v*(-NH) for 3-MBTSc and 4-NBTSc, respectively. A sharp peak between 1588–1599 cm^−1^ was assigned to *v*(C=N). Further, the peak between 1246–1343 cm^−1^ and 750–832 cm^−1^ are due to *v*(C=S). The presence of these peaks confirmed the linkage between the aldehydes and thiosemicarbazide indicating the formation of both the compounds. The peak at 1343 cm^−1^ corresponds to *v*(N=O). The obtained peaks are consistent with previous reports with minor changes [43,66,67]. The peaks between 1451–1454 cm^−1^ are due to the aromatic C=C stretching. Also, other peaks in the FTIR spectra of the ligands mainly fall in the fingerprint region.

^1^H-NMR spectra are recorded in d^6^-DMSO and presented in Figures S2 and S3 (Supplementary Information). In ^1^H-NMR spectra of 3-MBTSc, the prominent proton signal peaks at 8.19 ppm and 11.40 ppm were assigned to the hydrogen atom of NH_2_ and NH groups. The higher proton signals found for NH group (Figure S2A) could be due to the intermolecular hydrogen bonding [42]. Further, a proton signal peak at 3.80 ppm and between 6.95–7.44 ppm was due to –OCH_3_ and C-Ph groups, respectively, in ^1^H-NMR spectra of 3-MBTSc that were similar to the reported values [42]. Moreover, in ^1^H-NMR spectra of 4-NBTSc, NH_2_ and NH protons were assigned to 8.38 ppm and 11.66 ppm peaks respectively (Figure S3A). The obtained results were similar to those observed in ^1^H-NMR spectra of 2-nitrobenzaldehyde thiosemicarbazone [44]. The ^13^C-NMR spectrum of both compounds showed the carbon peaks at 178–179 ppm and 55.29 ppm for C=S and -OCH_3_ groups respectively (Figure S2B and Figure S3B). The C=N signal peak was observed at 159.6 ppm and 148.14 ppm for both 3-MBTSc and 4-NBTSc compounds, respectively. The signals for aromatic carbon atoms were observed in the range of 111–143 ppm which were corroborated with previous reports [44].

### 3.5. Anticancer Potential of 3-MBTSc and 4-NBTSc Compounds

**Figure 4:**
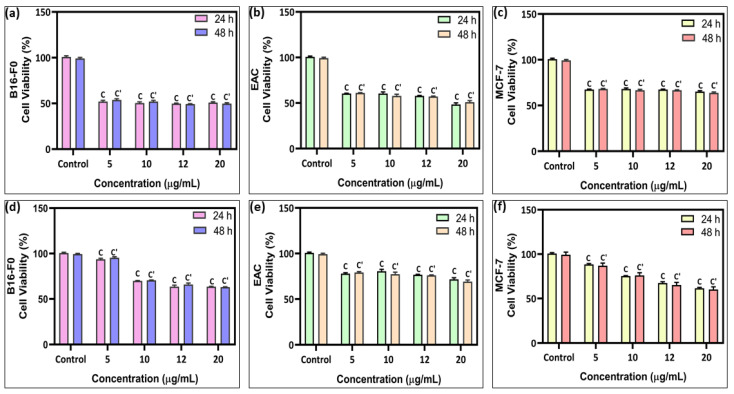
Anticancer potential of (**a**–**c**) 3-MBTSc and (**d**–**f**) 4-NBTSc compounds on B16-F0, EAC, and MCF-7 cells. The toxicity was measured by MTT in a dose-dependent manner till 48 h. All the experiments were conducted three independent times and each treatment was kept in triplicates (n = 3). c, c’ = *** *p* < 0.001.

Both 3-MBTSc and 4-NBTSc compounds were evaluated for their anticancer potential against MCF-7, B16-F0, and EAC cells in dose-dependent manner till 48 h. The IC_50_ values for 3-MBTSc compound on MCF-7, B16-F0, and EAC cells were calculated to be 2.821 ± 0.008 µg/mL, 2.904 ± 0.013 µg/mL, and 3.355 ± 0.012 µg/mL, respectively (Table 3). From the obtained results, the anticancer potential of 3-MBTSc compound was more potent against MCF-7 cells followed by B16-F0 and EAC cells (Figure 4a–c). Further, The IC_50_ values for 4-NBTSc compound on MCF-7, B16-F0, and EAC cells were calculated to be 7.102 ± 0.010 µg/mL, 7.129 ± 0.012 µg/mL, and 3.832 ± 0.014 µg/mL, respectively. From the obtained results, the anticancer potential of 4-NBTSc compound was more potent against EAC cells followed by MCF-7 and B16-F0 cells (Figure 4d–f, Table 3). Moreover, the 3-MBTSc compounds showed higher anticancer therapeutic effects compared to 4-NBTSc compound (Table 3). To validate the anticancer potential of both compounds, the DOX was used as a positive control. The calculated IC_50_ value of DOX on MCF-7 cells was 3.162 ± 0.018 µg/mL, respectively (Figure S4). 

### 3.6. Cytotoxicity Study of 3-MBTSc and 4-NBTSc Compounds

**Figure 5:**
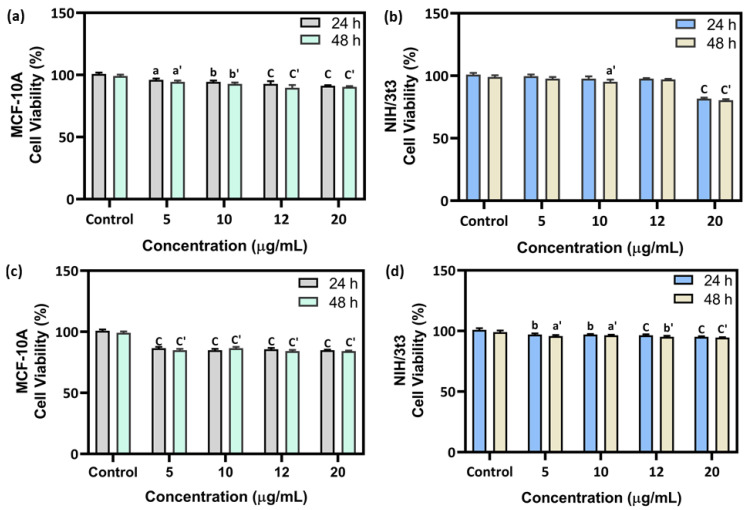
Cytotoxicity of (**a**,**b**) 3-MBTSc and (**c**,**d**) 4-NBTSc compounds on MCF-10A and NIH-3T3 cells. The toxicity was measured by MTT in a dose-dependent manner till 48 h. All the experiments were conducted three independent times and each treatment was kept in triplicates (*n* = 3). a, a’ = * *p* < 0.05; b, b’ = ** *p* < 0.01; c, c’ = *** *p* < 0.001.

The toxicity of both 3-MBTSc and 4-NBTSc compounds was analyzed by MTT test on MCF-10A and NIH-3T3 cells for 48 h. We observed that both compounds displayed more than 80 % viability on both cells and showed toxicity less than 20% indicating the biocompatible nature of both compounds with normal cells (Figure 5).

### 3.7. Effects of 3-MBTSc and 4-NBTSc Compounds on Gene Expression of B16-F0 Melanoma, EAC and MCF-7 Human Breast Cancer Cells

**Figure 6:**
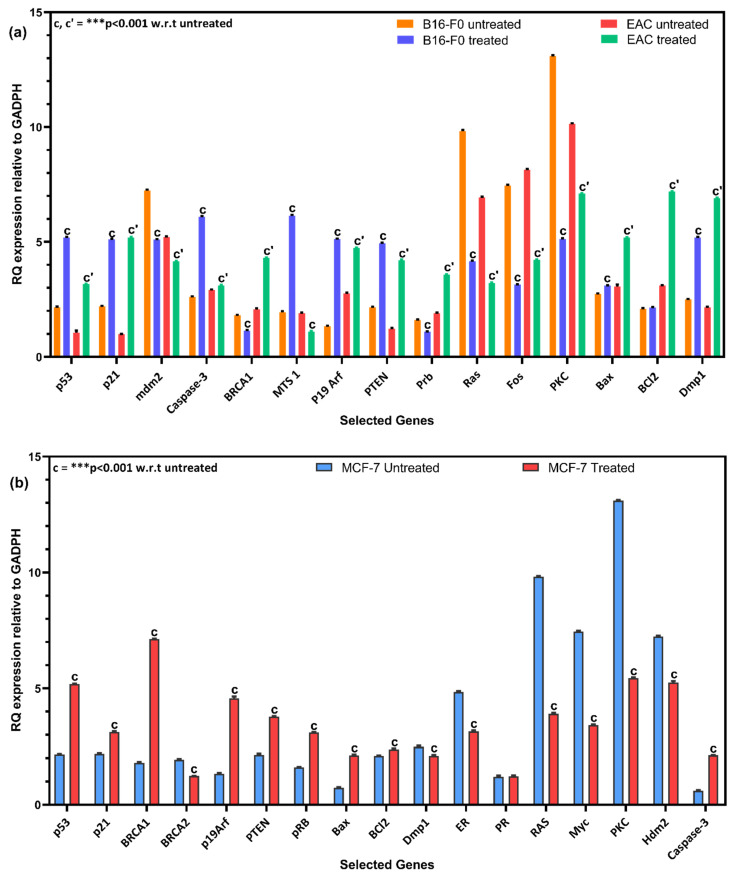
Selected gene expression after 3-MBTSc treatment (**a**) EAC and B16-F0 melanoma and (**b**) MCF-7 breast cancer.

**Figure 7:**
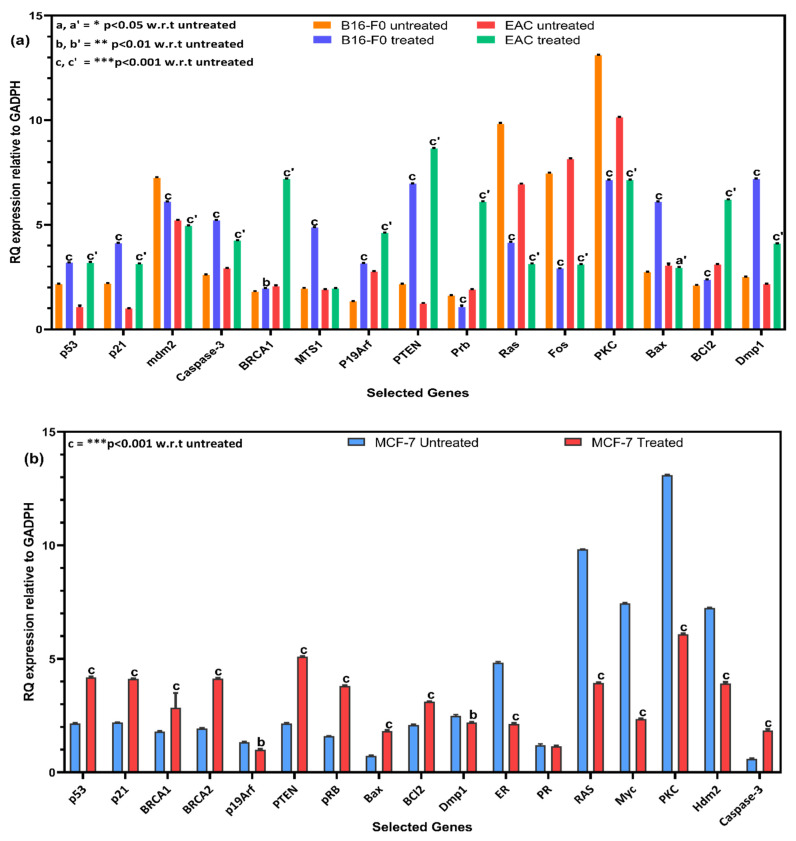
Effect of 4-NBTSc on gene expression (**a**) EAC and B16-F0 melanoma and (**b**) MCF-7 breast cancer.

The RT-qPCR was performed on RNA isolated from B16-F0 melanoma, EAC and MCF-7 human breast cancer cells treated with 3-MBTSc and 4-NBTSc. Interestingly, the gene expressions of treated cells are significantly different from untreated and control cells (*p* < 0.001) (Figure 6 and Figure 7 and Tables S4–S9). Tumor suppressor genes are upregulated, and oncogenes are downregulated significantly. The activity of both ligands varied with respect to cell line type. In general, 3-MBTSc treated cells showed a significant upregulation of BRCA1, Dmp1, and MTS1 in MCF-7, EAC, and B16-F0 cells, where they are upregulated in 5.33, 4.75, and 4.25 folds, respectively (Figure 6 and Tables S4–S6). The effect of the 3-MBTSc varied with respect to cell line and type of genes. In line with this, BRCA1, p19Arf, and p53 genes in MCF-7 breast cancer cells; MTS1, p19Arf, and p53 genes in B16-F0 melanoma, and Dmp1, p21, and BCl2 genes in EAC cell lines are among significantly upregulated tumor suppressor genes. On the contrary, with respect to untreated cells, the activities of oncogenes in 3-MBTSc treated cells are significantly (*p* < 0.001) downregulated. Along with this line, the expression of oncogenes like PKC, RAS, and Fos with −7.96, −5.66, and −4.31 folds in B16-F0 cells; PKC, RAS, and Myc with −7.64, −5.92, and −4.02 folds in MCF-7 tumor cells are significantly downregulated (Tables S4–S6). Conversely, the expression of Caspase-3, an apoptosis-inducing gene, is upregulated by 3.50 and 1.53 folds in B16-F0 melanoma and MCF-7 tumor cells, respectively.

Similarly, 4-NBTSc treated cells, most tumor suppressor genes showed a significant upregulation. Notably, PTEN, BRCA1, and pRB are highly activated with 7.42, 5.13, and 4.19 folds in EAC, respectively (Figure 7 and Tables S7–S9). Likewise, PTEN, Dmp1, and Bax are upregulated with 4.82, 4.7 and 3.35 folds in B16-F0 melanoma. Also, in MCF-7 breast cancer cell lines, PTEN, pRB, and BRCA2 are upregulated with 2.95, 2.21, and 2.195 folds, respectively. In comparison, the expressions of oncogenes are significantly downregulated in 4-NBTSc treated cells. In MCF-7 tumor cells, PKC, RAS, and Myc are downregulated by −7.0, −5.88 and −5.1 folds, respectively. Likewise, PKC, RAS, and Fos genes are downregulated with 5.95, 5.68, and 4.54 in B16-F0 cells. Similarly, Fos, RAS, and PKC are downregulated with 5.0, 3.81, and 3.0 folds in EAC cells, respectively (Tables S7–S9).

## 4. Discussion

The structure, substituents, cancer types, and the dose used have all been reported to influence the compound’s activity [68,69,70,71]. The IC_50_ and cell viability results from this study showed that the activities of the test compound vary depending on cell line type, concentration used, and substituent type. According to the results, the test compounds exhibited a good anticancer activity, with IC_50_ values less than 10 µg/mL. The lowest IC_50_ value of MCF-7 in our study is lower than previous reports for the standard drugs of cisplatin and carboplatin [72,73], and higher than Doxorubicin [74]. Also, it is lower than other bioactive molecules such as Organotin (IV) derivative, Diphenyl Sn (IV)) [73]. In contrast, the result is higher than a palladium (II) thiosemicarbazone complexes [75]. Moreover, the current result is comparable to the IC_50_ of pyrazole-naphthalene derivative bearing ethoxy at the 4-position of the phenyl ring [72].

Our study showed cell growth inhibition increased with respect to an increase in concentration of treatment increased. In line with this, the test compound showed a comparative cell growth inhibition activity with the standard drug Doxorubicin against MCF-7 breast cancer cell line. Theoretically, the highest IC_50_ is associated with the lowest anticancer activity and the lowest cell growth inhibition [76]. Likewise, in our study, the cell viability and the IC_50_ results support each other. Overall, the test compounds showed a significant (*p* < 0.05 *) growth inhibition activity indicating their therapeutic potential as anticancer agents for further study.

A well-known tumor suppressor gene, p53, plays an essential role in numerous signaling pathways to maintain genome integrity [77]. As a result, most tumorigenesis are associated with the mutation of p53 genes [78]. We found an upregulated expression of different tumor suppressor genes that interact with p53 in various signaling pathways in treated cells than control. Genes such as PTEN, a positive regulator p53 [79]; BRCA1, a co-activator of p53 [80,81]; and Dmp1, a transcription factor that interacts directly with p53 [82] are upregulated in 3-MBTSc and 4-NBTSc treated tumor cells than untreated. Also, p19Arf and p21, the transcriptional targets of p53, an inhibitor of cyclin-dependent kinases and Mdm2, are upregulated. In addition, pRB, one of the transcription regulators and a negative regulator of cell proliferation [83], is upregulated in treated MCF-7 and EAC tumor cells. Likewise, Caspase-3, a cysteine–aspartic acid protease that cleaves cellular targets and executes cell death [84] is upregulated in treated cell with both compounds. This could be associated with a potent pro-apoptotic effect of the test drug through p53-dependent pathways [85]. However, further detailed studies should be conducted to support this conclusion.

In contrast, a downregulated gene expressions are found in oncogenes. Among the tested oncogenes, PKC and RAS are highly downregulated in both 3-MBTSc and 4-NBTSc treated tumor cells. The interaction between p53 and RAS signaling regulates cancer chemoresistance. And p53 and RAS inversely regulate apoptosis through AKT- and ERK-mediated signaling pathways [86]. In our study, p53 is upregulated while RAS is downregulated in treated cell lines. Also, Mdm2, a potent negative regulator of p53 gene [78,87], and C Myc, transcriptional targets of p53 are downregulated. These results indicates that the test compounds can be a potential candidate to inhibit oncogenes expression.

The best binding modes of the docked ligand, 3-Methoxybenzaldehyde thiosemicarbazone (3-MBTSc) and 4-Nitrobenzaldehyde thiosemicarbazone (4-NBTSc) with PARP (PDB ID: 1A26), VEGFR-1 (PDB ID: 5ABD), TGF-β1 (PDB ID: 3RJR), BRAF^V600E^ (PDB ID: 6V34), are depicted in Figure 1 and Table 1. As presented in Table 1, 3-MBTSc interacted with the active site of 1A26 (Figure 1A) by forming two hydrogen bonding with Ser854 and Tyr 907 amino acid and released −32.04 KCal/Mol. Likewise, 4-NBTSc interacted with four amino acid residues of 1A26 (Figure 1B) and released −33.0 KCal/mol binding energy. The hydrogen atom of the compound interacted with the oxygen atom of Ser 681 amino acid and formed three hydrogen bonds. Also, two hydrogen bonds with the nitrogen atom of Lys 684, one hydrogen bond with each oxygen atom of Thr 866 and Met 675 are formed. Additionally, 3-MBTSc strongly interacted with 5ABD (Figure 1C) in three hydrogen bonds with Tyrosine 199 and released −30.33 KCal/mol of binding energy. Whereas, when 4-NBTSc interacted with 5ABD (Figure 1D), it has involved in six hydrogen bonding and released −22.33 KCal/mol of binding energy. The hydrogen atom of 4-NBTSc interacted with the oxygen atom of Pro 173, Asp 175, Tyr 199 to form one hydrogen bond with each amino acid. Simultaneously, the hydrogen atom of the test compound formed three hydrogen bonds with the nitrogen atom of Lysine at Lys 171 and Lys 200.

A strong interaction is observed when the test compound 3-MBTSc interacted with 3RJR (Figure 1E) and released −32.13 KCal/mol of energy. The hydrogen atom of the compound interacted with the oxygen atom of Leucine (Leu 218 and Leu 129) to form two hydrogen bonds. In comparison, the best interaction is found when 4-NBTSc extended into the active site of 3RJR which released −42.34 KCal/mol of energy. The hydrogen atom of the compound has interacted with the nitrogen atom of Ala 219 and Leu 218 amino acids and the oxygen atom of Ser 228 and Asp 217 amino acids (Figure 1F). On the other hand, Thr 589 amino acid is involved in making three hydrogen bonds with 3-MBTSc (Figure 1G). Similarly, the hydrogen atom of 4-NBTSc interacted with 6V34 and formed four hydrogen bonds through the oxygen atom of Threonine 589 amino acid (Figure 1H). A total of −30.69 and −29.46 KCal/mol of energy is released during the interaction of 6V34 with 3-MBTSc and 4-NBTSc, respectively. Besides, Glu 586 formed one hydrogen bond with 3-MBTSc through its oxygen atom.

The ADMET prediction revealed that both test compounds have high intestinal absorption and BBB+ property (permeable) but cannot penetrate Caco-2. When predicting the efflux by P-glycoprotein (P-gp), they prove to be both non-substrate and non-inhibitor. Cytochrome P450 enzymes are essential for the metabolism and elimination of drugs. Although there are many isoforms, 90% of drugs are metabolized by six of them, with CYP3A4 and CYP2D6 being the two most significant enzymes [88]. Both the test compounds exhibit non-substrate and non-inhibitor behavior on most of the CYP450 isoforms. However, 3-MBTSc inhibits CYP450 1A2 isoform, and 4-NBTSc inhibits CYP450 1A2 and CYP450 2C19 isoforms indicating that it may affect the activity of these enzymes during biotransformation of drugs. Moreover, both compounds presented low CYP inhibitory promiscuity properties. AMES toxicity and carcinogen results showed that the test compounds have mutagenic and non-carcinogenic natures. Chemical acute oral toxicity is an important endpoint in drug design [89]. Based on the acute oral toxicity (ADMET prediction profile), compounds are categorized into four groups. category I = LD50 ≤ 50 mg/kg, category II = 50 < LD50 ≤ 500 mg/kg, category III = 500 < LD50 ≤ 5000 mg/kg and category IV = LD50 > 5000 mg/kg [89]. In this study, acute oral toxicity of 3-MBTSc falls in Category III and 4-NBTSc in category II. Based on the admetSAR predicted LD50 dose in rat model 3-MBTSc has a better LD50 value than 4-NBTSc. Overall, the ADMET properties of the two compounds were very interesting, but 3-Methoxybenzaldehyde demonstrated a better ADMET property with less toxicity and less inhibition to normal cell function.

## 5. Conclusions

In this study, we have successfully synthesized 3-Methoxybenzaldehyde thiosemicarbazone and 4-Nitrobenzaldehyde thiosemicarbazone and characterized their structures using IR, ^1^HNMR and ^13^C NMR. The in vitro anticancer activities of the tested compounds are selective, substituent, dose- and cell line dependent. Tumor suppressor genes are upregulated more than seven-fold, and oncogenes are downregulated close to eight-fold more in treated tumor cells than untreated cells. The in silico computational study was consistent with the experimental work. The molecular docking showed that the test compounds strongly bind with the amino acid residue of the targeted proteins. The compounds have a favorable ADMET and drug-likeness profile. Overall, 3-MBTSc and 4-NBTSc have the potential to be multitargeting drug candidates for further studies. Moreover, 3-MBTSc has better activity than 4-NBTSc.

## Figures and Tables

**Table 1:** Molecular docking results of 3-MBTSc and 4-NBTSc with targeted proteins expressed in breast and skin cancers.

Compound (C)	Target Proteins (P)	Amino Acid (A.A)	Type of Hydrogen Bond Interaction (Compound-Protein Complex) (C∙∙∙A.A of P)	Binding Free Energy (K Cal/mol)	Bond Length (A^0^)
3-MBTSc	1A26	Ser 854	N-H---O-H	−32.04	2.702
		Tyr 907	S-H---O=C		2.5665
4-NBTSc	1A26	Lys 684	N-H---N-H	−33.0	3.380
		Lys 684	N-H---N-H		2.7266
		Thr 866	N-H---O-H		3.4682
		Ser 681	N-H---O-H		2.5093
		Ser 681	N-H---O-H		3.2158
		Ser 681	N-H---O-H		2.3759
		Met 675	S-H---O=C		3.0379
3-MBTSc	5ABD	Tyr 199	N-H---O-H	−30.33	2.9587
		Tyr 199	N-H---O-H		2.6311
		Tyr 199	N-H---O-H		2.8478
4-NBTSc	5ABD	Pro 173	S-H---O=C	−22.33	2.3968
		Asp 175	S-H---O=C		3.385
		Lys 171	N-H---N-H		3.3874
		Lys 171	N-H---N-H		2.8633
		Tyr 199	O-H---O=C		3.1201
		Lys 200	N-H---N-H		3.1493
3-MBTSc	3RJR	Leu 218	S-H---O=C	−32.13	2.6683
		Leu 129	S-H---O=C		3.4729
4-NBTSc	3RJR	Ala 219	N-H---N-H	−42.34	3.5020
		Asp 217	-S-H---O=C		2.6237
		Leu 218	N-H---N-H		3.1209
		Ser 228	N-H---O-H		3.3725
3-MBTSc	6V34	Glu 586	N-H---O=C	−30.69	3.5768
		Thr 589	N-H---O-H		2.5020
		Thr 589	H-N---O-H		3.0345
		Thr 589	N-H---O-H		2.3331
4-NBTSc	6V34	Thr 589	N-H---O-H	−29.46	3.1137
		Thr 589	H-N---O-H		2.7631
		Thr 589	N-H---O-H		2.7257
		Thr 589	H-N---O-H		3.2469

**Table 2:** Comparison of drug-likeness and medicinal chemistry properties of 3-MBTSc and 4-NBTSc from OSIRIS Property Explorer and SwissADME.

Properties	3-MBTSc	4-NBTSc
Molecular weight	209.27 g/mol	224.24 g/mol
clog P	1.46	0.61
Solubility log S	−2.92	−3.36
No. of rotatable bonds	4	4
No. of H-bond acceptors	2	3
No. of H-bond donors	2	2
TPSA	91.73 Å²	128.3 Å²
Molar Refractivity	59.69	62.02
Drug likeness	Yes	Yes
Lipinski rule of five (RO5) violation	0 violation	0 violation
Drug score	0.96	0.45
Bioavailability Score	0.55	0.55
Synthetic accessibility	2.13	2.28

**Table 3:** IC_50_ values of 3-MBTSc and 4-NBTSc compounds on different cancer cells.

Cell Lines	IC_50_ Values	
	3-MBTSc	4-NBTSc
MCF-7	2.821 ± 0.008	7.102 ± 0.010
B16-F0	2.904 ± 0.013	7.129 ± 0.012
EAC	3.355 ± 0.012	3.832 ± 0.014

## Supplementary Materials

The following are available online at https://www.mdpi.com/article/10.3390/biomedicines9101375/s1. Figure S1: Bioavailability Radar of (A) 3-MBTSc and (B) 4-NBTSc compounds; Figure S2: (A) ^1^H-NMR and (B) ^13^C-NMR spectra of 3-MBTSc; Figure S3: (A) ^1^H-NMR and (B) ^13^C-NMR spectra of 4-NBTSc; Figure S4: Anticancer effect of DOX on MCF-7 cells as a positive control. The toxicity was measured by MTT in a dose-dependent manner till 48 h. All the experiments were conducted three independent times and each treatment was kept in triplicates (n = 3). C, C’ = *** *p* < 0.001; Table S1: List of genes and primers used in MCF-7 and MCF-10A cells; Table S2: List of primers used in EAC, B16-F0 and NIH-3T3 cells; Table S3: ADMET properties of the test compound from admetSAR; Table S4: Effect of 3-MBTSc on expression of different genes in EAC cells; Table S5: Effect of 3-MBTSc on genes expression in B16-F0 cells; Table S6: Effect of 3-MBTSc on different genes expression in MCF-7 cells; Table S7: Effect of 4-NBTSc on selected genes expression in EAC cells; Table S8: Effect of 4-NBTSc on genes expression in B16-F0 cells; Table S9: Effect of 4-NBTSc on genes expression in MCF-7 cells.
