# Supplementary material for: Synthesis, In Silico Study, and Anti-Cancer Activity of Thiosemicarbazone Derivatives
Source: Biomedicines. 2021 Oct 1;9(10):1375. doi: 10.3390/biomedicines9101375 (PMC8533299; doi:10.3390/biomedicines9101375)
Supplement: Supplementary file 1 [file biomedicines-09-01375-s001.zip › biomedicines-1385375-supplementary.pdf]

Supplementary Information

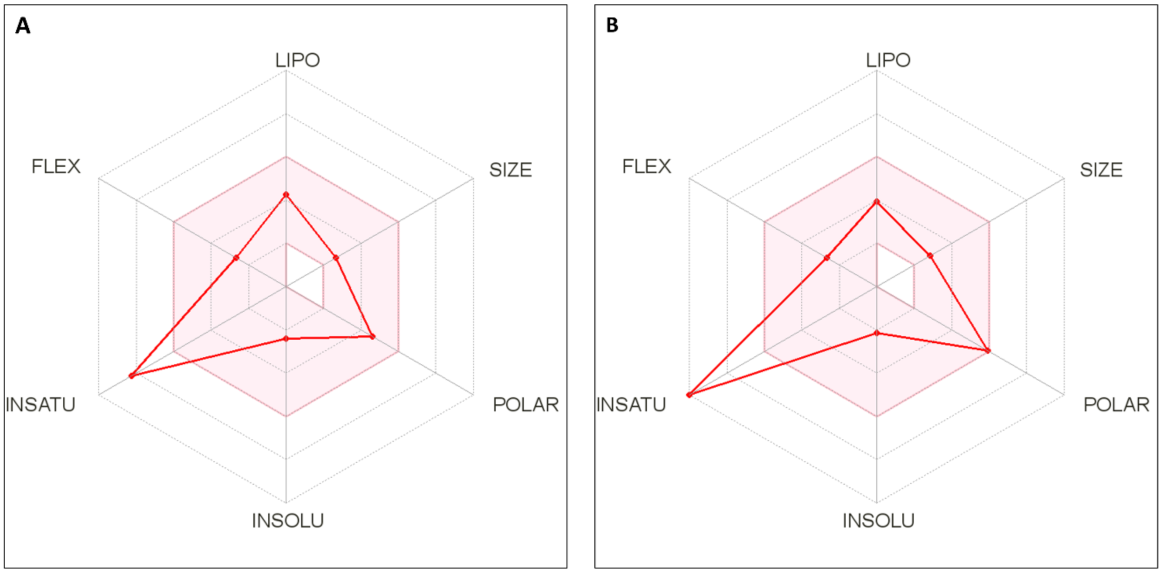

Figure S1. Bioavailability Radar of A) 3-MBTSc and B) 4-NBTSc compounds.

The Bioavailability Radar enables a first glance at the drug-likeness of a molecule**.** The pink area represents the optimal range for each property (lipophilicity: XLOGP3 between -0.7 and +5.0, size: MW between 150 and 500 g/mol, polarity: TPSA between 20 and 130 Å2, solubility: log *S* not higher than 6, saturation: fraction of carbons in the sp3 hybridization not less than 0.25, and flexibility: no more than 9 rotatable bonds.

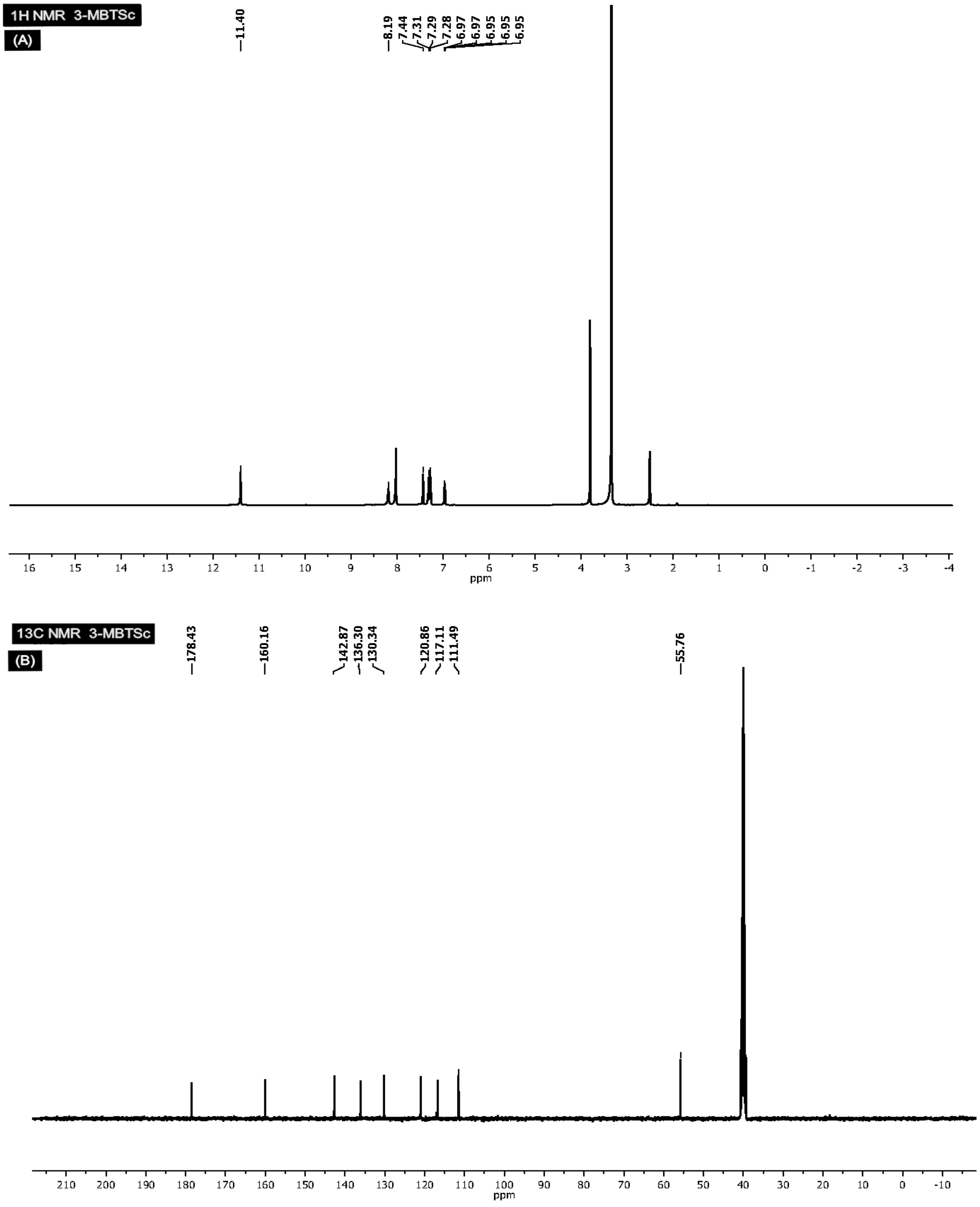

Figure S2. **(A)** ^{1}H-NMR and **(B)** ^{13}C-NMR spectra of 3-MBTSc.

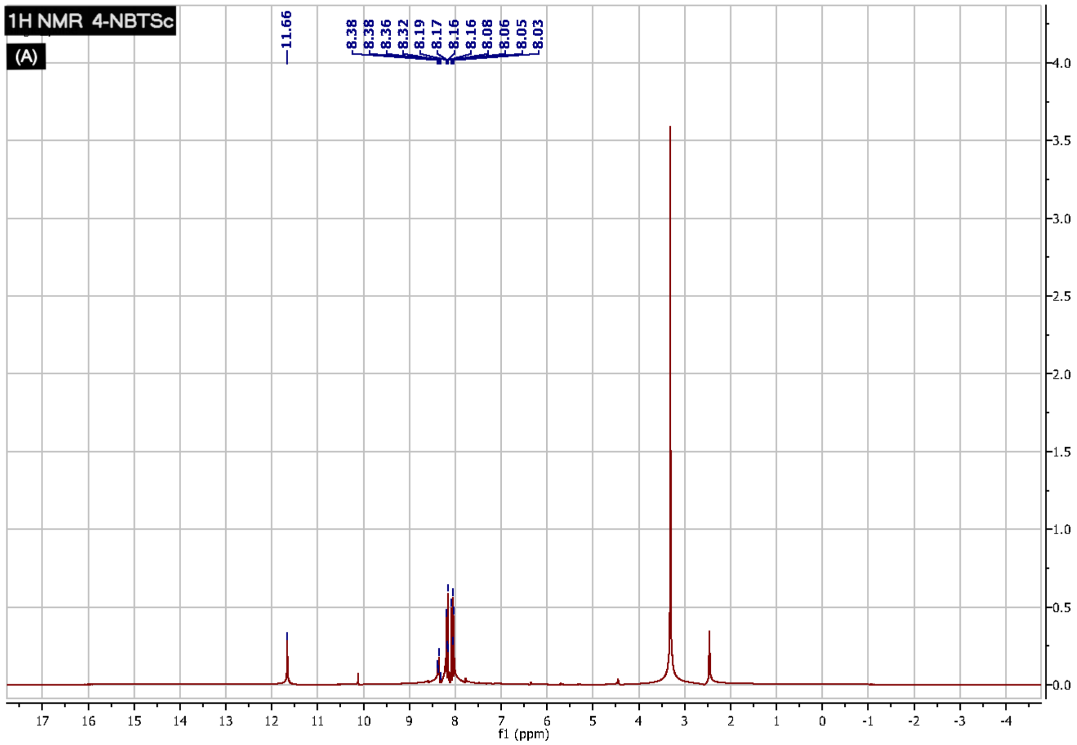

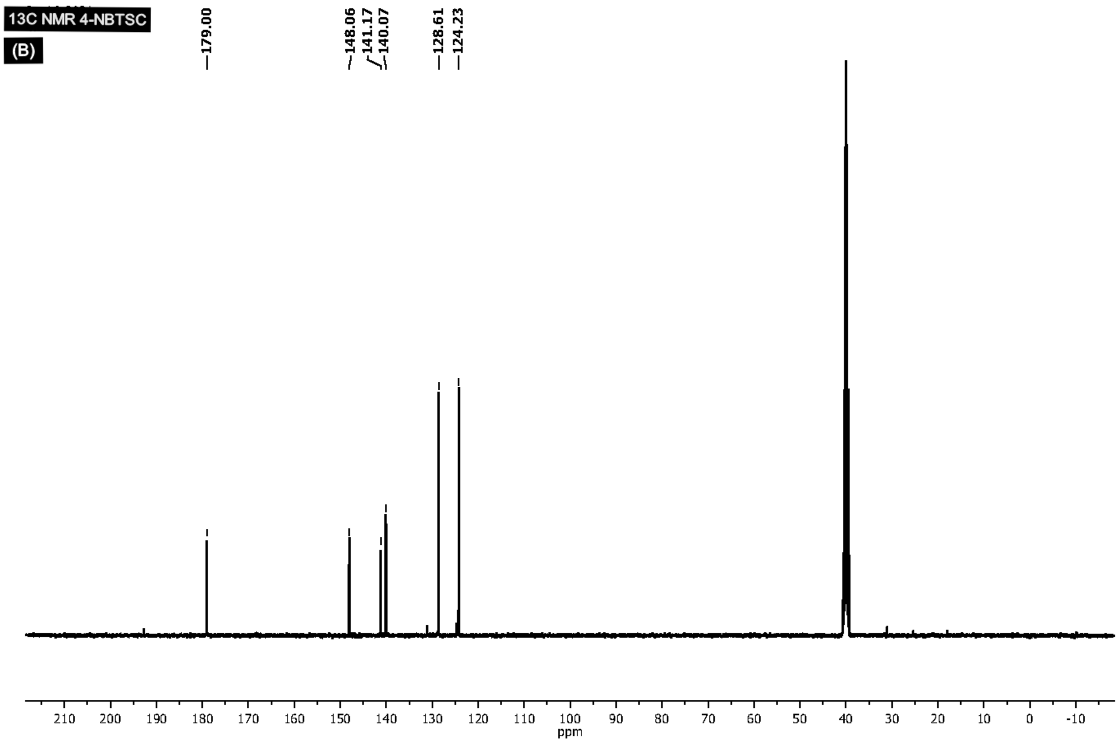

Figure S3. **(A)** ^{1}H-NMR and **(B)** ^{13}C-NMR spectra of 4-NBTSc.

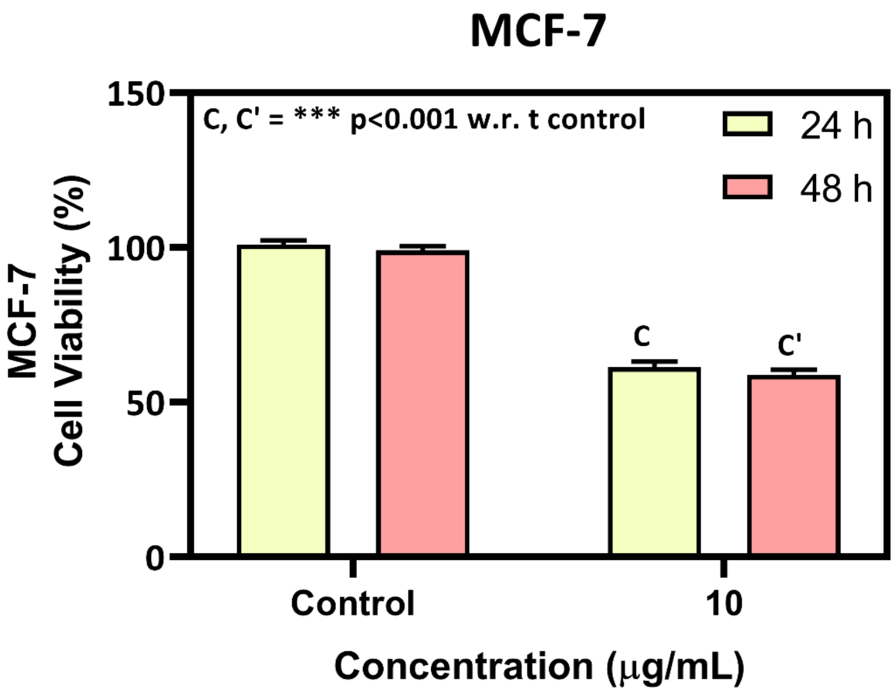

Figure S4. Anticancer effect of DOX on MCF-7 cells as a positive control. The toxicity was measured by MTT in a dose-dependent manner till 48 h. All the experiments were conducted three independent times and each treatment was kept in triplicates (n = 3). C, C′ = *** $p < 0.001$

Table S1. List of genes and primers used in MCF-7 and MCF-10A cells.

S. No.	Gene-mRNA/primers	Primer Sequence	Forward & Reverse
**1**	*Ras*	GTTGGAGCTGGTGGCGTAGG	F
		GCCCTCCCCAGTCCTCATGT	R
**2**	*Caspase 3*	AGGCCTGCCGTGGTACAGAA	F
		GCATGGCACAAAGCGACTGG	R
**3**	*Myc*	CGTTGCGGTCACACCCTTCT	F
		CTCTGAGACGAGCTTGGCGG	R
**4**	*GADPH*	CAATGCCTCCTGCACCACCA	F
		TCCACCACTGACACGTTGGC	R
**5**	*P53*	CACCAGCAGCTCCTACACCG	F
		TCAACCCACAGCTGCACAGG	R
**6**	*P21(CDKN1A)*	ACTGGAGGGTGACTTCGCCT	F
		TCCACCTGGGGACCCTTCAG	R
**7**	*P14 (CDKN2A)*	GGGGTCGGGTAGAGGAGGTG	F
		CCACCAGCGTGTCCAGGAAG	R
**8**	*hdm2*	CCAGCTTCGGAACAAGAGACCC	F
		AGCTTGGCACGCCAAACAAA	R
**9**	*BRCA1*	GGGCCACACGATTTGACGGA	F
		GAGCAGCAGCTGGACTCTGG	R
**10**	*BRCA2*	TCCACACCTGTCTCAGCCCA	F
		GCCACAACTCCTTGGTGGCT	R
**11**	*PTEN*	TCCCAGTCAGAGGCGCTATGT	F
		TTGTCTTCCCGTCGTGTGGG	R
**12**	*pRb*	ACCTCAGCCTTCCAGACCCA	F
		GGGTGCTCAGACAGAAGGCG	R
**13**	*ER*	CCGACCAGATGGTCAGTGCC	F
		ATCCACAAAGCCTGGCACCC	R
**14**	*PR*	TCCACGTGCCTATCCTGCCT	F
		GAGGGTACGCGTCGTCCTTG	R
**15**	*BAX*	GGGCCCTTTTGCTTCAGGGT	F
		CACGGCGGCAATCATCCTCT	R
**16**	*BCL2*	GGGCCCTTTTGCTTCAGGGT	F
		CACGGCGGCAATCATCCTCT	R
**17**	*Dmp1 (hDMTF1)*	ACTGGGCAACAATAGGGGCG	F
		ACAGCTGCCCAAGACACACC	R
**18**	*PKC*	CAGCGGCGGCTTCTACATCA	F
		GCCTCTGGAGACATCGTGCC	R

Table S2. List of primers used in EAC, B16-F0 and NIH-3T3 cells.

S. No.	Gene-mRNA/primers	Primer Sequence	Forward & Reverse
**1**	*Ras*	GTTGGAGCTGGTGGCGTAGG	F
		AGCCCTCCCCAGTTCTCATGT	R
**2**	*Caspase 3*	CGGAGCTGGACTGTGGCATT	F
		GCTTGTGCGCGTACAGCTTC	R
**3**	*MTS1 (CDKN2A) Tr-1*	CTGCGAGGACCCCACTACCT	F
		ACACAAAGACCACCCAGCGG	R
**4**	*GAPDH*	TGTGGATGGCCCCTCTGGAA	F
		TGACCTTGCCCACAGCCTTG	R
**5**	*P53*	TTCGTGTTTGTGCCTGCCCT	F
		TGGAGTGAGCCCTGCTGTCT	R
**6**	*P21(CDKN1A) Tr-2*	CGACCTGGGAGGGGACAAGA	F
		GGGGAATCTTCAGGCCGCTC	R
**7**	*P19 (CDKN2A) first*	CTGCGAGGACCCCACTACCT	F
		ACACAAAGACCACCCAGCGG	R
**8**	*mdm2*	CTGAGGGAGATGTGCAGCGG	F
		GAGAGCTCGTGCCCTTCGTC	R
**9**	*BRCA1*	GGAGCTTCCATCGCTCACCC	F
		GCATCCAGATCCCGACACCG	R
**10**	*Fos*	AGGTCTGCCTGAGGCTTCCA	F
		TGTGCAGAGGCTCCCAGTCT	R
**11**	*PTEN*	GCTGGAAAGGGACGGACTGG	F
		TGAAACAGCAGTGCCACGGG	R
**12**	*pRb*	CGCCTTCTGTCTGACCACCC	F
		GGGCAGCGTGAGGAAGATCC	R
**13**	*BAX*	TCCAGGATCGAGCAGGGAGG	F
		GTCCGTGTCCACGTCAGCAA	R
**14**	*BCL2*	GGGATGCTGGAGATGCGGAC	F
		TGCGAAGTCACGACGGTAGC	R
**15**	*Dmp1*	GCTGCCGGCTGATGAAGGAT	F
		GACGATGTCACCTGGCTCCG	R
**16**	*PKC*	GGGCTACCGGATGCCTTGTC	F
		GCAGGTACTCGAAGGTGGGC	R

Table S3. ADMET properties of the test compound from admetSAR.

Property	Test Compounds	
	**3-MBTSc**	**4-NBTSc**
**Absorption**		
Blood-Brain Barrier	BBB+	BBB+
Human Intestinal Absorption	HIA+	HIA+
Caco-2 Permeability	Caco2-	Caco2-
P-glycoprotein Substrate/ Inhibitor	Non-substrate/ non-inhibitor	Non-substrate/ non-inhibitor
Renal Organic Cation Transporter	Non-inhibitor	Non-inhibitor
**Distribution**		
Subcellular localization	Mitochondria	Mitochondria
**Metabolism**		
CYP450 2C9 Substrate	Non-substrate	Non-substrate
CYP450 2D6 Substrate	Non-substrate	Non-substrate
CYP450 3A4 Substrate	Non-substrate	Non-substrate
CYP450 1A2 Inhibitor	Inhibitor	Inhibitor
CYP450 2C9 Inhibitor	Non-inhibitor	Non-inhibitor
CYP450 2D6 Inhibitor	Non-inhibitor	Non-inhibitor
CYP450 2C19 Inhibitor	Non-inhibitor	Inhibitor
CYP450 3A4 Inhibitor	Non-inhibitor	Non-inhibitor
CYP Inhibitory Promiscuity	Low CYP Inhibitory Promiscuity	Low CYP Inhibitory Promiscuity
**Excretion and Toxicity**		
AMES Toxicity	AMES toxic	AMES toxic
Carcinogens	Non-carcinogens	Non-carcinogens
Fish Toxicity	High FHMT	High FHMT
Tetrahymena Pyriformis Toxicity	High TPT	High TPT
Honey Bee Toxicity	High HBT	Low HBT
Biodegradation	Not readily biodegradable	Not readily biodegradable
Acute Oral Toxicity	III	II
Carcinogenicity (Three-class)	Non-required	Non-required
**ADMET Predicted Profile --- Regression**		
**Absorption**		
Aqueous solubility (LogS)	-2.5224	-2.6772
Caco-2 Permeability (LogPapp, cm/s)	0.5998	0.9655
**Toxicity**		
Rat Acute Toxicity (LD50, mol/kg)	2.0271	2.7674
Fish Toxicity (pLC50, mg/L)	1.5020	1.6612
Tetrahymena Pyriformis Toxicity (pIGC50, μg/L)	0.7449	0.6522

Table S4. Effect of 3-MBTSc on expression of different genes in EAC cells.

Name of gene	Untreated (RQ value)		Treated (RQ value)	Fold Change (EAC treated-(EAC untreated)	P-value (P<0.05)
	NIH-3T3	EAC	EAC		
p53	1.075±0.007	1.06±0.085	3.16±0.028	2.1 upregulated	
p21	0.995±0.007	0.995±0.007	5.19±0.028	4.195 upregulated	
Mdm2	1.065±0.092	5.205±0.035	4.145±0.035	-1.06 downregulated	
Caspase-3	1.005±0.007	2.9±0.028	3.095±0.035	0.195 upregulated	
BRCA1	0.985±0.021	2.06±0.057	4.305±0.021	2.245 upregulated	
MTS 1	1.01±0.014	1.905±0.021	1.1±0.028	-0.805 downregulated	
p19 Arf	1.06±0.085	2.75±0.042	4.73±0.028	1.98 upregulated	
PTEN	1.01±0.014	1.23±0.028	4.205±0.035	2.975 upregulated	
pRB	1.045±0.064	1.905±0.021	3.575±0.021	1.67 upregulated	
RAS	1.005±0.007	6.94±0.028	3.215±0.021	-3.725 downregulated	
Fos	0.955±0.064	8.14±0.042	4.22±0.014	-3.92 downregulated	
PKC	1.02±0.028	10.135±0.035	7.105±0.021	-3.03 downregulated	
Bax	1.035±0.049	3.05±0.099	5.195±0.021	2.145 upregulated	
BCl2	1.02±0.028	3.09±0.028	7.195±0.021	4.105 upregulated	
Dmp1	1.055±0.078	2.15±0.028	6.9±0.028	4.75 upregulated	
					< 0.0001****

GAPDH was the housekeeping gene and used for RQ analysis

Table S5. Effect of 3-MBTSc on genes expression in B16-F0 cells.

Name of gene	Untreated (RQ value)		Treated (RQ value)	Fold Change (B16-F0 treated-(B16-F0 untreated)	P- value (P<0.05)
	NIH/3t3	B16-F0	B16-F0		
p53	1.075±0.007	2.16±0.028	5.185±0.035	3.025 upregulated	
p21	0.995±0.007	2.19±0.028	5.115±0.021	2.925 upregulated	
Mdm2	1.065±0.092	7.24±0.042	5.105±0.021	-2.135 downregulated	
Caspase-3	1.005±0.007	2.6±0.028	6.105±0.021	3.505 upregulated	
BRCA1	0.985±0.021	1.8±0.028	1.14±0.028	-0.660 downregulated	
MTS 1	1.01±0.014	1.935±0.049	6.14±0.042	4.205 upregulated	
p19 Arf	1.06±0.085	1.335±0.021	5.125±0.021	3.790 upregulated	
PTEN	1.01±0.014	2.145±0.035	4.94±0.028	2.795 upregulated	
pRB	1.045±0.064	1.6±0.028	1.105±0.007	-0.495 downregulated	
RAS	1.005±0.007	9.825±0.049	4.16±0.028	-5.665 downregulated	
Fos	0.955±0.064	7.45±0.042	3.135±0.021	-4.315 downregulated	
PKC	1.02±0.028	13.095±0.035	5.13±0.028	-7.965 downregulated	
Bax	1.035±0.049	2.735±0.021	3.095±0.021	0.360 upregulated	
BCl2	1.02±0.028	2.09±0.028	2.14±0.028	0.050 upregulated	
Dmp1	1.055±0.078	2.49±0.028	5.19±0.028	2.700 upregulated	
					0.0006***

GAPDH was the housekeeping gene and used for RQ analysis

Table S6. Effect of 3-MBTSc on different genes expression in MCF-7 cells.

Name of gene	Untreated (RQ value)		Treated (RQ value)	Fold Change (MCF 7 treated-(MCF 7 untreated)	P- value (P<0.05)
	MCF-10A	MCF-7	MCF-7		
p53	1.06 ± 0.06	2.16 ± 0.02	5.185 ± 0.015	3.025 upregulated	
p21	0.995 ± 0.005	2.19 ± 0.02	3.125 ± 0.025	0.935 upregulated	
BRCA1	0.985 ± 0.015	1.8 ± 0.02	7.13 ± 0.01	5.33 upregulated	
BRCA2	1.01 ± 0.01	1.94 ± 0.035	1.235 ± 0.005	-0.7 downregulated	
p19 Arf	1.06 ± 0.06	1.34 ± 0.015	4.575 ± 0.055	3.24 upregulated	
PTEN	1.01 ± 0.01	2.145 ± 0.025	3.78 ± 0.02	1.635 upregulated	
pRB	1.045 ± 0.045	1.6 ± 0.02	3.11 ± 0.01	1.51 upregulated	
Bax	1.035 ± 0.035	0.73 ± 0.025	2.125 ± 0.025	1.4 upregulated	
BCl2	1.02 ± 0.02	2.09 ± 0.02	2.375 ± 0.025	0.285 upregulated	
Dmp1	1.055 ± 0.055	2.49 ± 0.02	2.1 ± 0.03	-0.39 upregulated	
ER	1.015 ± 0.005	4.84 ± 0.04	3.15 ± 0.03	-1.69 upregulated	
PR	0.995 ± 0.005	1.195 ± 0.015	1.23 ± 0.02	0.035 upregulated	
RAS	1.005 ± 0.005	9.83 ± 0.035	3.905 ± 0.035	-5.92 upregulated	
Myc	0.955 ± 0.045	7.45 ± 0.03	3.43 ± 0.015	-4.02 downregulated	
PKC	1.02 ± 0.02	13.1 ± 0.025	5.455 ± 0.015	-7.64 downregulated	
Hdm2	1.065 ± 0.065	7.24 ± 0.03	5.26 ± 0.045	-1.98 downregulated	
Caspase-3	1.005 ± 0.005	0.6 ± 0.02	2.13 ± 0.01	1.53 upregulated	
					0.0011**

GAPDH was the housekeeping gene and used for RQ analysis

Table S7. Effect of 4-NBTSc on selected genes expression in EAC cells.

Name of gene	Untreated (RQ value)		Treated (RQ value)	Fold Change (EAC treated-(EAC untreated)	P-value (P<0.05)
	NIH/3t3	EAC	EAC		
p53	1.075±0.007	1.06±0.085	3.165±0.064	2.105 upregulated	
p21	0.995±0.007	0.995±0.007	3.125±0.035	2.13 upregulated	
Mdm2	1.065±0.092	5.205±0.035	4.955±0.021	-0.25 downregulated	
Caspase-3	1.005±0.007	2.9±0.028	4.23±0.028	1.33 upregulated	
BRCA1	0.985±0.021	2.06±0.057	7.195±0.021	5.135 upregulated	
MTS 1	1.01±0.014	1.905±0.021	1.945±0.035	0.04 upregulated	
p19 Arf	1.06±0.085	2.75±0.042	4.605±0.021	1.855 upregulated	
PTEN	1.01±0.014	1.23±0.028	8.65±0.028	7.42 upregulated	
pRB	1.045±0.064	1.905±0.021	6.095±0.035	4.19 upregulated	
RAS	1.005±0.007	6.94±0.028	3.13±0.028	-3.81 downregulated	
Fos	0.955±0.064	8.14±0.042	3.095±0.021	-5.045 downregulated	
PKC	1.02±0.028	10.135±0.035	7.13±0.028	-3.005 downregulated	
Bax	1.035±0.049	3.05±0.099	2.945±0.035	-0.105 downregulated	
BCl2	1.02±0.028	3.09±0.028	6.195±0.021	3.105 upregulated	
Dmp1	1.055±0.078	2.15±0.028	4.1±0.014	1.95 upregulated	
					<0.0001****

GAPDH was the housekeeping gene and used for RQ analysis

Table S8. Effect of 4-NBTSc on genes expression in B16-F0 cells.

Name of gene	Untreated (RQ value)		Treated (RQ value)	Fold Change (B16-F0 treated-(B16-F0 untreated)	P value (P<0.05)
	**NIH/3t3**	**B16-F0**	**B16-F0**		
p53	1.075±0.007	2.16±0.028	3.185±0.021	1.025 upregulated	
p21	0.995±0.007	2.19±0.028	4.12±0.014	1.93 upregulated	
Mdm2	1.065±0.092	7.24±0.042	6.1±0.014	-1.14 downregulated	
Caspase-3	1.005±0.007	2.6±0.028	5.215±0.021	2.615 upregulated	
BRCA1	0.985±0.021	1.8±0.028	1.94±0.028	0.14 upregulated	
MTS 1	1.01±0.014	1.935±0.049	4.865±0.021	2.93 upregulated	
p19 Arf	1.06±0.085	1.335±0.021	3.145±0.035	1.81 upregulated	
PTEN	1.01±0.014	2.145±0.035	6.97±0.014	4.825 upregulated	
pRB	1.045±0.064	1.6±0.028	1.06±0.085	-0.54 downregulated	
RAS	1.005±0.007	9.825±0.049	4.145±0.035	-5.68 downregulated	
Fos	0.955±0.064	7.45±0.042	2.905±0.021	-4.545 downregulated	
PKC	1.02±0.028	13.095±0.035	7.14±0.028	-5.955 downregulated	
Bax	1.035±0.049	2.735±0.021	6.09±0.028	3.355 upregulated	
BCl2	1.02±0.028	2.09±0.028	2.36±0.028	0.27 upregulated	
Dmp1	1.055±0.078	2.49±0.028	7.19±0.028	4.7 upregulated	
					0.0003***

GAPDH was the housekeeping gene and used for RQ analysis

Table S9. Effect of 4-NBTSc on genes expression in MCF-7 cells.

Name of gene	Untreated (RQ value)			Fold Change (MCF 7 treated-(MCF 7 untreated)	P value (P<0.05)
	**MCF-10A**	**MCF-7**	**MCF-7**		
p53	1.06 ± 0.06	2.16 ± 0.02	4.185 ± 0.025	2.025 upregulated	
p21	0.995 ± 0.005	2.19 ± 0.02	4.12 ± 1.51	1.93 upregulated	
BRCA1	0.985 ± 0.015	1.8 ± 0.02	2.86 ± 0.455	1.06 upregulated	
BRCA2	1.01 ± 0.01	1.94 ± 0.035	4.13 ± 0.02	2.19 upregulated	
p19 Arf	1.06 ± 0.06	1.335 ± 0.015	0.995 ± 0.025	-0.34 downregulated	
PTEN	1.01 ± 0.01	2.145 ± 0.025	5.09 ± 0.02	2.945 upregulated	
pRB	1.045 ± 0.045	1.6 ± 0.02	3.81 ± 0.02	2.21 upregulated	
Bax	1.035 ± 0.035	0.725 ± 0.025	1.825 ± 0.025	1.1 upregulated	
BCl2	1.02 ± 0.02	2.09 ± 0.02	3.11 ± 0.01	1.02 upregulated	
Dmp1	1.055 ± 0.055	2.49 ± 0.02	2.19 ± 0.02	-0.3 downregulated	
ER	1.01 ± 0.01	4.84 ± 0.04	2.14 ± 0.03	-2.7 downregulated	
PR	0.995 ± 0.005	1.2 ± 0.015	1.15 ± 0.02	-0.05 downregulated	
RAS	1.005 ± 0.005	9.83 ± 0.035	3.94 ± 0.02	-5.89 downregulated	
Myc	0.955 ± 0.045	7.45 ± 0.03	2.35 ± 0.025	-5.1 downregulated	
PKC	1.02 ± 0.02	13.1 ± 0.025	6.09 ± 0.02	-7.01 downregulated	
Hdm2	1.065 ± 0.065	7.24 ± 0.03	3.92 ± 0.045	-3.32 downregulated	
Caspase-3	1.005 ± 0.005	0.6 ± 0.02	1.85 ± 0.04	1.25 upregulated	
					**0.0013****

GAPDH was the housekeeping gene and used for RQ analysis
